# CCL18-mediated down-regulation of miR98 and miR27b promotes breast cancer metastasis

**DOI:** 10.18632/oncotarget.4107

**Published:** 2015-05-12

**Authors:** Xiaorong Lin, Lijun Chen, Yandang Yao, Ruihua Zhao, Xiuying Cui, Jun Chen, Kailian Hou, Mingxia Zhang, Fengxi Su, Jingqi Chen, Erwei Song

**Affiliations:** ^1^ Guangdong Provincial Key Laboratory of Malignant Tumor Epigenetics and Gene Regulation, Sun Yat-Sen Memorial Hospital, Sun Yat-Sen University, Guangzhou, People's Republic of China; ^2^ Breast Tumor Center, SunYat-Sen Memorial Hospital, SunYat-Sen University, Guangzhou, People's Republic of China; ^3^ Department of Medical Oncology, No. 2 Affiliated Hospital, Guangzhou Medical University, Guangzhou, People's Republic of China; ^4^ Diagnosis and Treatment Center of Breast Diseases, Shantou Hospital, SunYat-Sen University, Shantou, Guangdong Province, People's Republic of China; ^5^ Department of Breast Tumor, The Third Hospital of Nanchang, Jiangxi Province, People's Republic of China; ^6^ Department of Oncology and Institute of Clinical Medicine, The First Affiliated Hospital of Zhengzhou University, Zhengzhou City, Henan Province, People's Republic of China

**Keywords:** miR98, miR27b, microRNA, CCL18, tumor associated macrophages, breast cancer

## Abstract

Our previous work has indicated that CCL18 secreted by tumor-associated macrophages (TAMs) promotes breast cancer metastasis, which is associated with poor patient prognosis. However, it remains unclear whether microRNAs (miRNAs), which may modulate multiple cellular pathways, are involved in the regulation of CCL18 signaling and the ensuing metastasis of breast cancer. In this study, we demonstrated that CCL18 reduces miR98 and miR27b expression via the N-Ras/ERK/PI3K/NFκB/Lin28b signaling pathway, while down-regulation of these mRNAs feedbacks to increase N-Ras and Lin28b levels. This cascade of events forms a positive feedback loop that sustains the activation of CCL18 signaling. More importantly, reduction in miR98 and miR27b enhances the epithelial-mesenchymal transition (EMT) of breast cancer cells, and thus promotes breast cancer metastasis. These findings suggest that down-regulation of miR98 and miR27b promotes CCL18-mediated invasion and migration of breast cancer cells.

## INTRODUCTION

Tumor-associated macrophages (TAMs) are the most abundant hematopoietic infiltrates in the microenvironment of breast cancer, and play an important role in the metastasis and progression of this malignancy [1]. TAMs in breast cancer are mostly skewed toward M2 polarization via alternative activation and can promote the invasiveness of breast cancer cells through the secretion of cytokines [2]. We have demonstrated that the presence of chemokine (C-C motif) ligand 18 (CCL18), a hallmark of M2 macrophages, promotes breast cancer metastasis through binding to its receptor PITPNM3 on breast cancer cells; this chemokine is also associated with a poor prognosis of patients with breast cancer [3]. We have also demonstrated that CCL18 induces the epithelial-mesenchymal transition (EMT) in breast cancer via activation of the NFκB pathway [4]. Therefore, CCL18 signaling is critical to the interaction between breast cancer cells and their microenvironment, which leads to breast cancer metastasis.

MiRNAs are a class of endogenous small non-coding RNA molecules that are approximately 22 nucleotides in length and repress mRNA translation and/or degrade mRNA molecules via binding to their 3′-untranslated regions (3′-UTR) [5]. MiRNAs may act as oncogenes or tumor suppressors via modulating multiple cellular pathways for proliferation, differentiation, apoptosis and invasion. As the most notable tumor-suppressing miRNAs, the let-7/miR98 family inhibits the expression of members of the Ras gene family and thus deactivates the downstream MAPK and/or PI3K/AKT signaling pathways, which results in suppressing the proliferation and invasion of breast cancer cells [6]. Another tumor-suppressing miRNA termed miR27b has also been shown to repress growth, angiogenesis and metastasis in many tumors, such as NCCL cancer, colorectal cancer and neuroblastoma [7-9]. However, the functional roles of miR27b in different sub-types of breast cancer remain controversial [10-12].

It has been noted that cytokines produced in the inflammatory milieu of cancer may decrease the expression of miRNAs in tumor cells, thus maintaining or dampening the activation of signal transduction pathways that mediate the cellular response [13]. For instance, IL-6, a pro-inflammatory cytokine that is abundant in the tumor stroma, induces EMT in colorectal cancer cells by phosphorylating STAT3 (signal transducer and activator of transcription 3). Furthermore, IL-6-induced down-regulation of miR34a maintains STAT3 activation [14]. Similarly, transforming growth factor (TGF)-β, an immune suppressing cytokine in the microenvironment of breast cancer, markedly reduces the expression of members of the miR200 family in breast cancer cells and thus relieves their suppression to ZEB1 and ZEB2, which underlies the mechanisms for TGF-β-induced EMT and tumor metastasis [15].

Our previous study showed that CCL18, the most abundant chemokine produced by TAMs in breast cancer, promotes breast cancer metastasis. However, it remains unclear whether miRNAs are involved in CCL18 signaling pathway in breast cancer cells to promote tumor metastasis. Our present study has shown that CCL18 reduces the expression of miR98 and miR27b in breast cancer cells via the N-Ras/ERK/PI3K/AKT/NFκB/Lin28b signaling cascade. Moreover, down-regulation of miR98 and miR27b maintains the activation of CCL18 signaling and promotes EMT and invasiveness of breast cancer cells.

## RESULTS

### CCL18 secreted by breast TAM reduces miR98 and miR27b expression

**Figure 1:**
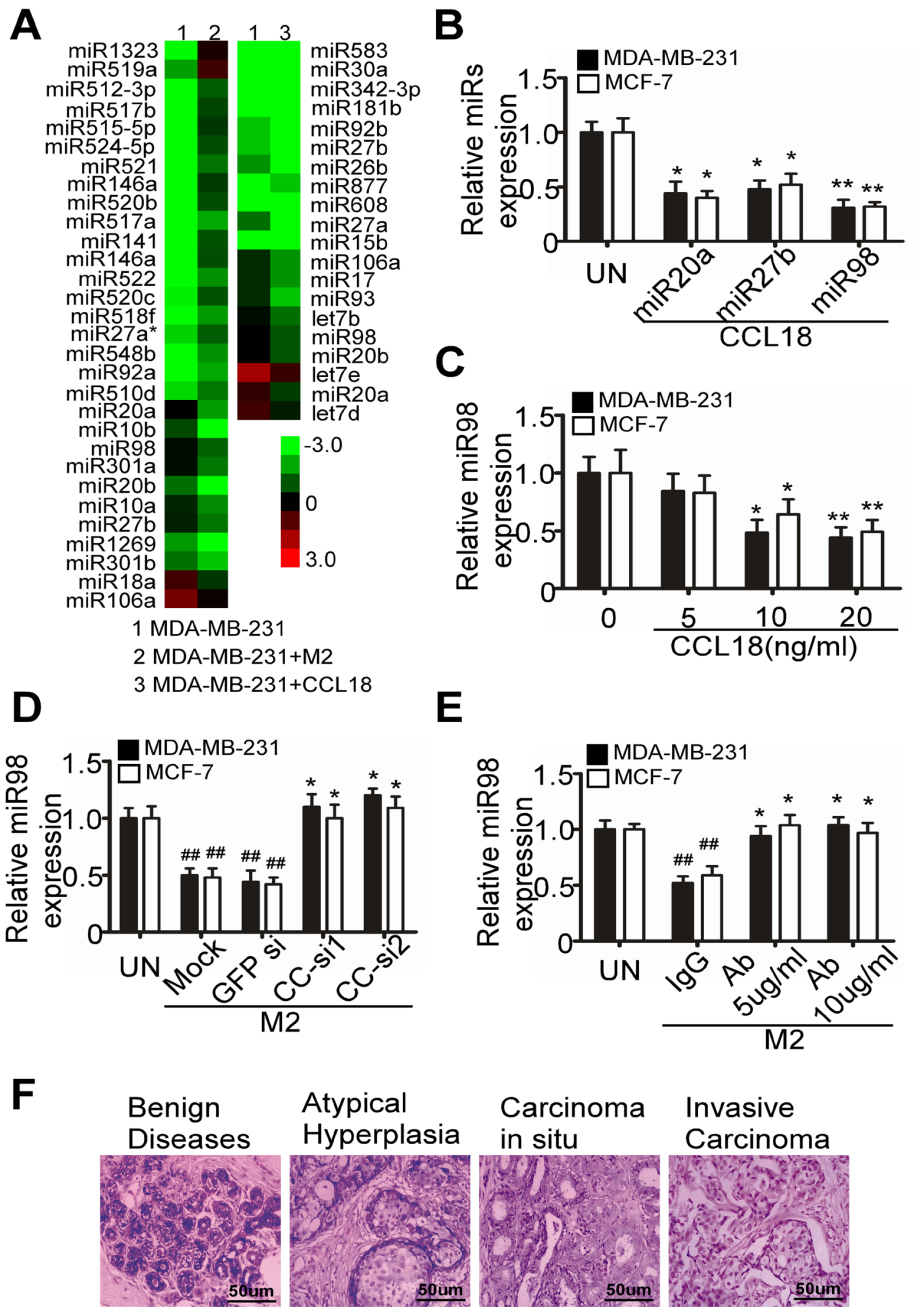
M2 macrophages decrease the expression of miR98 via CCL18 in breast cancer cells **A.** MiRNA microarray analysis showing that miRNAs were differentially expressed in MDA-MB-231 cells that were co-cultured with or without M2 macrophages and treated or not treated with CCL18. **B.** Alterations in miR20a, miR27b, and miR98 expression in MDA-MB-231 and MCF-7 breast cancer cells were determined by qRT-PCR (normalized to U6) after exposure to CCL18 (20 ng/ml) for 24 hr. **C.** The level of miR98 was normalized to U6 expression in breast cancer-derived MDA-MB-231 and MCF-7 cells that were treated with different concentrations of CCL18 for 24 hr, as assayed by qRT-PCR. For **B. C.**, the data expressed are representative of three independent experiments. *P < 0.05 and ** *P* < 0.01 compared with the untreated cells. Error bars correspond to the mean ± S.D. of triplicate experiments. **D.** and **E.** miR98 expression in MDA-MB-231 and MCF-7 cells that were co-cultured with M2 macrophages, which were mock-transfected or transfected with GFP-siRNA or either of the two CCL18-siRNAs **D.**, or exposed to isotype-matched IgG (10μg/ml), or different concentrations of the CCL18-neutralizing antibodies **E.**, as assayed by qRT-PCR. Data expressed are representative of three independent experiments. *P > 0.05 and ^##^ *P* < 0.01 compared with the untreated cells. Error bars correspond to the mean ± S.D. of triplicate experiments. **F.** miR98 expression in benign cystic fibrosis of the breast, atypical hyperplasia, breast carcinoma in situ, and invasive breast carcinoma, as assayed by in situ hybridization. miR98 signal stained blue in the cytoplasm.

To determine the effect of CCL18 on miRNA expression in breast cancer cells, we performed miRNA microarray analysis for MDA-MB-231 breast cancer cell line 24 hr after exposure to CCL18 or co-cultured with IL-4 stimulated M2 macrophages. As compared with the untreated cells, the expression of 20 miRNAs were significantly changed in MDA-MB-231 cells treated with 20 ng/ml CCL18, including 5 elevated and 15 reduced miRNAs by more than 2-fold. Additionally, when MDA-MB-231 cells were co-cultured with M2 macrophages induced by 45 ng/ml interleukin-4 (IL-4) for 24 hr, 19 miRNAs were increased and 11 were decreased by more than 2-fold. Among the altered miRNAs, miR98, miR20 and miR27b were consistently down-regulated by CCL18 and M2 macrophages (Figure 1A), and marked reduction in miR98 and miR27b was further confirmed by quantitative RT-PCR (qRT-PCR) in CCL18 (20 ng/ml)-treated MCF-7 and MDA-MB-231 cells (Figure 1B).

To further validate that CCL18 reduces the expression of the above miRNAs in breast cancer cells, we performed qRT-PCR to determine the levels of miR98 and miR27b in MCF-7 and MDA-MB-231 cells after treatment with CCL18 at various concentrations for 24 hr. In this assay, miR98 was decreased by CCL18 in a concentration-dependent manner (*P* < 0.05) (Figure 1C), whereas miR27b was not reduced until the concentration of CCL18 reached 20 ng/ml (*P* < 0.05) (Figure SA).

Additionally, to investigate whether M2 cells reduced the expression of miR98 and miR27b via CCL18, we co-cultured IL-4 stimulated M2 macrophages with MDA-MB-231 or MCF-7 cells in Boyden transwell chambers with 0.4 μm inserts for 24 hr. This system enables cytokine but not cellular communication between the upper and lower chambers. Marked decrease in miR98 expression in breast cancer cells that were co-cultured with M2 macrophages (*P* < 0.05) was determined by qRT-PCR (Figure 1D, 1E). However, M2 macrophages that were transfected with CCL18 siRNAs or treated with CCL18- neutralizing antibodies lost their ability to reduce miR98 expression in both breast cancer cell lines (*P* < 0.05) (Figure 1D, 1E). Therefore, M2 macrophages repress miR98 expression via CCL18.

Our previous study demonstrated that CCL18-positive TAMs are abundant in invasive breast cancer but are absent in benign breast tissue [3]. Here, we used *in situ* hybridization (ISH) to detect the expression of miR98 in the breast tissue of 100 patients (20 with benign disease, 20 with atypical hyperplasia, 20 with carcinoma *in situ*, and 40 with invasive carcinoma), and found that miR98 expression was significantly decreased in ductal carcinomas *in situ* (DCIS) and was further reduced in invasive breast cancer. By contrast, miR98 expression was abundant in all benign breast tissues, including fibrocystic lesions with or without atypical epithelial hyperplasia (Figure 1F). Therefore, high CCL18^+^ TAM infiltration may correlate with low miR98 expression in breast cancer, which is in agreement with the *in-vitro* data.

### MiR98 was down-regulated at post-transcriptional level by CCL18-induced Lin28b

**Figure 2:**
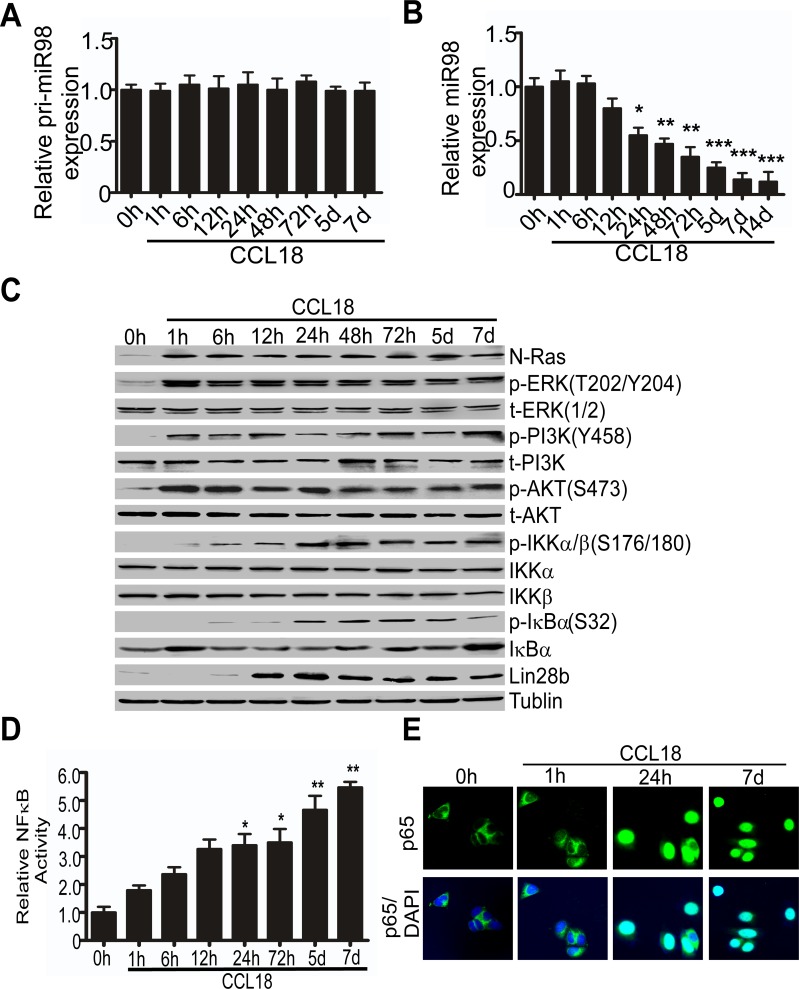
CCL18 reduces the expression of miR98 at the post-transcriptional level via the N-Ras/ERK/PI3K/NFκB/Lin28b pathway **A.** and **B.** qRT-PCR analysis of pri-miR98 **A.** or miR98 **B.** expression in MCF-7 cells with or without CCL18 (20 ng/ml) treatment at different time points. Data are representative of three independent experiments. *P < 0.05, **P < 0.01 and ***P < 0.001 compared with the untreated group (0 hr). Error bars correspond to the mean ± S.D. of triplicate experiments. **C.** Western blot of MCF-7 cells untreated (0 hr) or treated with CCL18 (20 ng/ml) at different time points for the expression of the phosphorylated forms and total protein expression of N-Ras, ERK, PI3K, AKT, IKKα, IKKβ, IκBα, and Lin28b; tubulin was used as the loading control. **D.** A relative luciferase assay detected the activity of NFκB in MCF-7 cells exposed to CCL18 (20 ng/ml) for different periods of time. Data are representative of three independent experiments. *P < 0.05 and **P < 0.01 compared with the untreated group (0 hr). Error bars correspond to the mean ± S.D. of triplicate experiments. **E.** Confocal fluorescence microscopy of p65/DAPI staining in MCF-7 cells untreated (0 hr) or treated with CCL18 (20 ng/ml) for 1 hr, 24 hr, or 7 d.

To investigate whether CCL18 reduces miR98 expression at transcriptional or post-transcriptional level, we employed qRT-PCR to detect the primary and mature miR98 expression in MCF-7 breast cancer cells treated with CCL18 (20 ng/ml) for 1 hr to 14 days (d). Although primary miR98 expression was not significantly changed following CCL18 treatment for 1 hr to 7 d (Figure 2A), mature miR98 was decreased in a time-dependent manner after treated with CCL18 (Figure 2B). Similarly, mature miR27b expression was decreased in a time-dependent manner after exposure to CCL18 from 12 to 48 hr (Figure SB). These finding indicates that CCL18 persistently suppresses the expression of mature miR98 and miR27b in breast cancer cells.

To investigate the mechanisms for CCL18-induced miR98 reduction, we performed western blot analysis for the activation of CCL18-related Ras/ERK/PI3K/Lin28b signaling pathway at various time points. Our previous studies showed that CCL18 activates the PyK2-Src-FAK-ERK/PI3K signaling cascade in breast cancer cells [3, 16], while N-Ras and Lin28b locate upstream and downstream, respectively, of this cascade. We found that the expression of N-Ras protein was increased in MCF-7 breast cancer cells that were treated with CCL18 for 1 hr, and the phosphorylation of ERK at Thr202 and Tyr204, PI3K P85 at Tyr458 and Akt at Ser473 was concurrently induced and remained active for 7 d (Figure 2C). Since NFκB may transcribe Lin28b that inhibits the expression of let-7/miR98 family members by interacting with the let-7 precursor loop [17], we suspected that CCL18-activated NFκB might also increase Lin28b expression and lead to miR98 reduction [16]. In the MCF-7 cells that were treated with CCL18 for 6 hr to 7 d, IκBα total protein was reduced along with the phosphorylation of IKKα/β at Ser176/180 and that of IκBα at Ser32 (Figure 2C). NFκB activity was therefore induced and escalated over time to a 5-fold increase on the 7th day, which was measured by transient transfection with an NFκB-dependent luciferase reporter (Figure 2D). Moreover, we found that CCL18 induced nuclear translocation of p65 at 24 hr and 7 d, but not at 0 hr or 1 hr, which indicates that the activation of the NFκB pathway was induced by CCL18 (Figure 2E). Additionally, Lin28b expression was increased at 12 hr and remained elevated until 7 d, which was consistent with the down-regulation of mature miR98 (Figure 2C). These data indicate that CCL18 reduces miR98 levels while it activates the Ras/ERK/PI3K/NFκB/Lin28b pathway.

**Figure 3:**
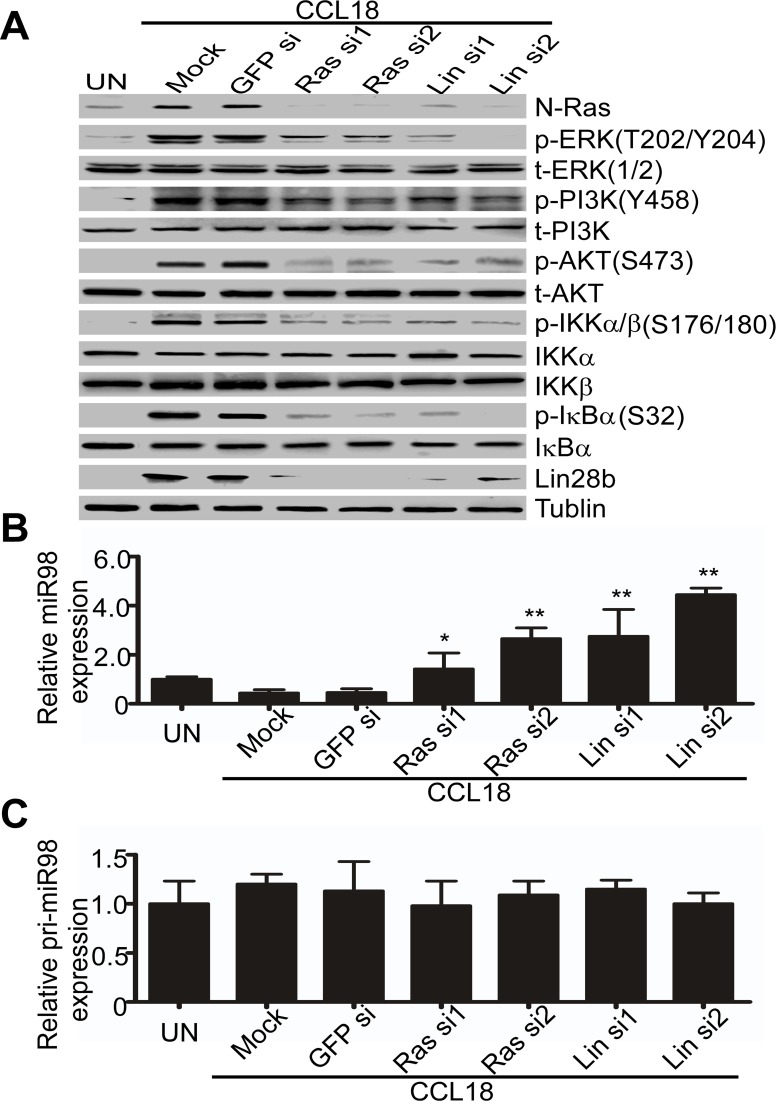
Integrity of the N-Ras/ERK/PI3K/NFκB/Lin28b pathway is necessary for CCL18 to down-regulate miR98 **A.**Western blot for phosphorylated and total protein expression of N-Ras/ERK/PI3K/NFκB/Lin28b in MCF-7 cells that were untreated (UN), mock-transfected, or transfected with N-Ras or Lin28b-siRNAs or with GFP-siRNA in the presence or absence of CCL18. **B.** and **C.** Similar to **A.**, qRT-PCR analysis of mature miR98 **B.** or pri-miR98 **C.** expression in MCF-7 cells with or without the addition of CCL18 (20 ng/ml). Data are representative of three independent experiments. *P < 0.05 and ** *P* < 0.01 compared with the untreated group. Error bars correspond to the mean ± S.D. of triplicate experiments.

To further investigate whether the integrated N-Ras/ERK/PI3K/NFκB/Lin28b signaling pathway was essential for the repression of miR98 by CCL18, we transfected siRNAs targeting N-Ras or Lin28b mRNA into MCF-7 breast cancer cells, which were then exposed to CCL18 (20 ng/ml) for 48 hr. Western blot analysis showed that compared with the mock- or GFP-siRNA-transfected cells, the CCL18-induced phosphorylation of ERK/PI3K/AKT/IKKα/β/IκBα was inhibited and the entire signaling pathway was inactivated when the expression of N-Ras or Lin28b was silenced (Figure 3A). Moreover, qRT-PCR data showed that CCL18 upregulated mature miR98 by 1.5-2.5-fold or 2.7-4.4-fold in N-Ras- or Lin28b-silenced cells, but not in mock- or GFP-siRNA-transfected cells (Figure 3B). However, no changes were observed in pri-miR98 (Figure 3C). Together, these results suggest that mature miR98 is down-regulated by CCL18 through a persistently activated N-Ras/ERK/PI3K/AKT/NFκB/Lin28b pathway.

### Reduced miR98 and miR27b sustain CCL18 signaling by targeting to N-Ras and Lin28b

**Figure 4:**
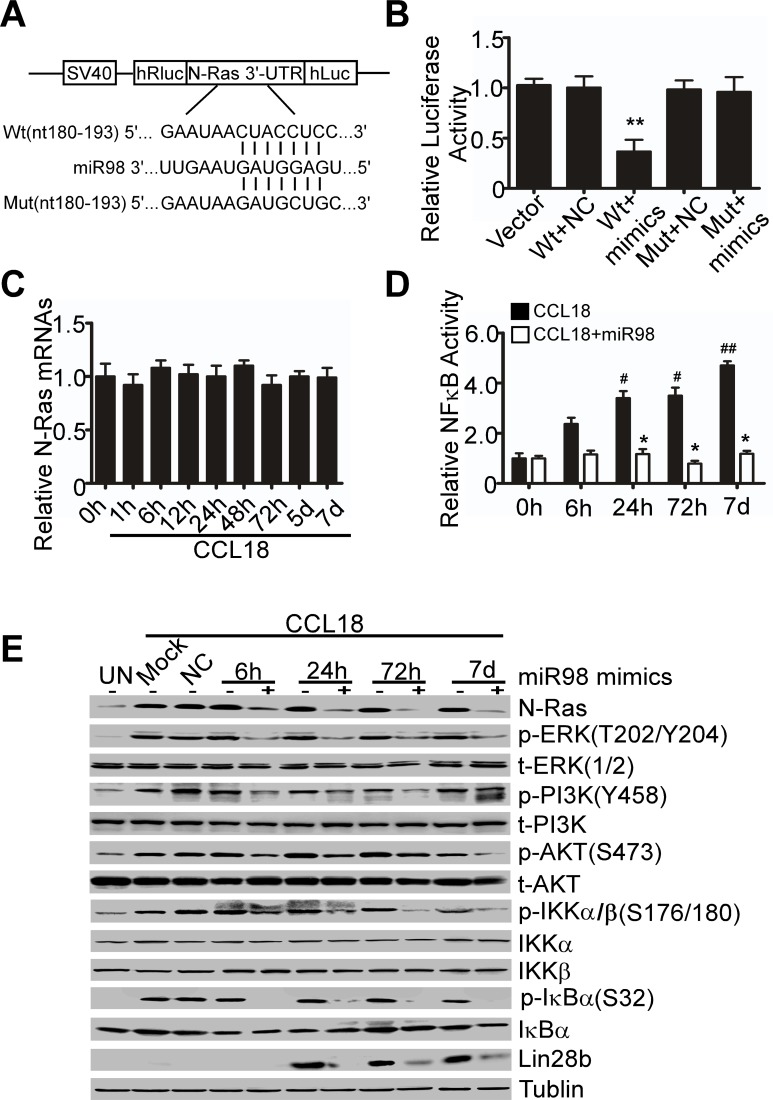
MiR98 targets to N-Ras 3′UTR to inhibit the activation of intracellular CCL18 signaling pathway **A.** The miR98 targeting sequence located in the 3′-UTR of N-Ras. **B.** Dual-luciferase reporter assays in 293T cells that were transiently transfected with the reporter plasmids (pmiR-control, pmiR-N-Ras-Wt or pmiR-N-Ras -Mut) along with a hRluc/hluc expression plasmid. Luciferase activities were measured in 293T cells that were transfected with miR98 for 24 hr and normalized against hluc values. The data are the mean ± S.D. of separate transfections (*n* = 3) and are shown as the ratio of the activity of Renilla reniformis luciferase to the activity of firefly luciferase. The percentage of relative luciferase (RLU) activity was then plotted. **P < 0.01 compared with the vector-only group. **C.** qRT-PCR analysis of N-Ras mRNA expression in MCF-7 cells untreated (0 hr) or treated with CCL18 (20 ng/ml) for different periods of time. **D.** NFκB-dependent luciferase reporter assay in miR98-transfected or non-transfected MCF-7 cells that were stimulated by CCL18 (20 ng/ml) for different periods of time. Data are representative of three independent experiments. ^#^*P* < 0.05 and ^##^ *P* < 0.01 for comparison of NFκB activity in cells that were treated with CCL18 to that in untreated cells (0 hr). *P > 0.05 for comparison of NFκB activity in cells that were treated with CCL18 and transfected with miR98 mimics to that in untreated cells (0 hr). Error bars correspond to the mean ± S.D. of triplicate experiments. **E.** Western blot analysis for the phosphorylated and total protein expression of N-Ras, ERK, PI3K, AKT, IKKα, IKKβ, IκBα, and Lin28b in MCF-7 cells transfected with or without miR98 mimics; tubulin was used as the loading control.

It has been established that the let-7/miR98 family plays an important role in tumor suppression mainly by targeting to Ras oncogenes [6]. Here, we predicted that the 3′-UTR of N-Ras harbored a putative miR98 binding site with 3 miRNA target algorithms (Pictar, Targetscan and miRanda). To confirm whether the N-Ras-3′-UTR was a functional target of miR98, we cloned the N-Ras 3′-UTR fragment that contained a putative miR98 binding site into the luciferase reporter vector (Figure 4A). Luciferase activity assays demonstrated that miR98 mimics, but not the negative control, reduced the luciferase activity by nearly 3-fold in 293T cells transfected with N-Ras-wt, compared with those transfected with N-Ras-mut or with empty vector (Figure 4B). These data suggest that miR98 specifically binds to the 3′-UTR region of N-Ras and reduces N-Ras expression. To determine whether miR98 reduces N-Ras expression at post-transcriptional level, we used qRT-PCR and western blotting to examine N-Ras mRNA and protein expression, respectively, upon stimulation by CCL18 from 1 hr to 7 d. No change was detected in N-Ras at the mRNA level, whereas the expression of N-Ras protein was steadily elevated (Figure 2C, 4C, 4E). These results suggest that miR98 regulates N-Ras expression at the post-transcriptional level. To further analyze whether miR98 feedbacks to regulate the ERK/PI3K/NFκB/Lin28b pathway by targeting to N-Ras, we transfected miR98 mimics into MCF-7 cells, which were then stimulated by CCL18 for 6 hr, 24 hr, 72 hr or 7 d. Western blot analysis indicated that transfection with miR98, but not the mock or negative control, inhibited the activation of the N-Ras/ERK/PI3K/NFκB/Lin28b signaling pathway induced by CCL18 in breast cancer cells (Figure 4E). Additionally, an NFκB-dependent luciferase reporter assay demonstrated that miR98 suppressed NFκB activation induced by CCL18 from 6 hr to 7 d (Figure 4D). Taken together, these data suggest that reduction in miR98 increases the expression of N-Ras, which is upstream to ERK/PI3K, to sustain the activation of the N-Ras/ERK/PI3K/NFκB/Lin28b signaling pathway in a positive feedback manner.

To study whether the down-regulation of miR27b acts synergistically with miR98 to regulate the ERK/PI3K/NFκB/Lin28b pathway, we employed the miRNA target algorithms and predicted that Lin28b was the potential target of miR27b. Luciferase reporter plasmids that contained the wild-type 3′-UTR sequence of Lin28b or a mutant sequence were transfected into 293T cells with miR27b mimics or the negative control (NC) (Figure SD). We found that the Lin28b luciferase activity of the wild-type reporter was reduced by 2-fold upon miR27b over-expression (Figure SE), whereas the Lin28b luciferase activity of the mutant reporter showed no change in response to miR27b over-expression (Figure SE). These results indicate that the 3′-UTR of Lin28b harbors a binding site for miR27b. More importantly, transfection with miR27b mimics, but not with the negative control or untransfection, decreased the protein level of Lin28b and suppressed the activation of ERK/PI3K/NFκB/Lin28b pathway upon CCL18 treatment (Figure SC). Collectively, these results suggest that miR98 and miR27b are involved in the positive feedback loop of CCL18 signaling pathway to sustain its activation.

### Reduction in miR98 and miR27b enhances CCL18-induced EMT and invasiveness of breast cancer cells

**Figure 5:**
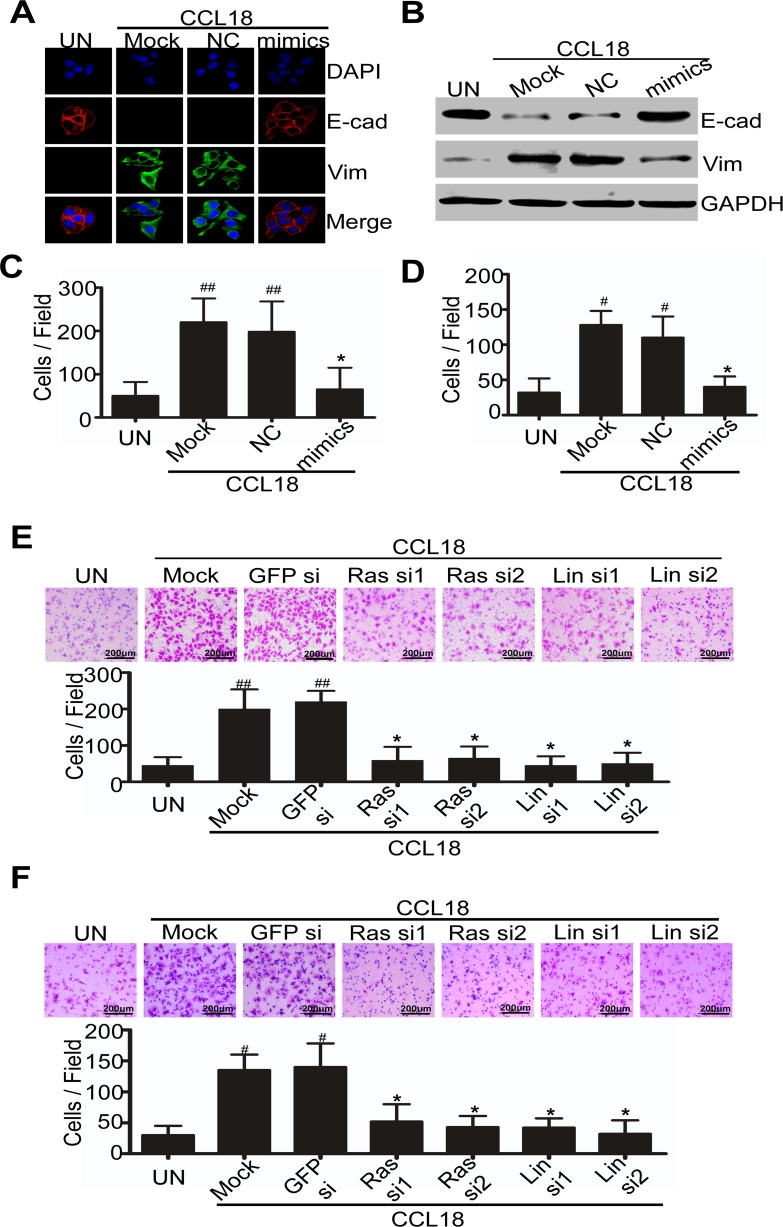
CCL18 reduces miR98 expression to enhance EMT, migration and invasion of breast cancer cells **A. and B.** Immunoﬂuorescence staining **A.** and western blot **B.** detected E-cadherin and vimentin in MCF-7 cells that were transfected with miR98 mimics or with a negative control and in cells that were mock-transfected followed by treatment with CCL18 (20 ng/ml) for 7 d. **C.** and **D.** Boyden chamber assay for MDA-MB-231 cells that were transiently transfected with miR98 mimics, negative control or with mock transfection and then plated on 8 μm cell culture inserts coated with **D.** or without **C.** matrigel, and treated with medium only or with CCL18 (20 ng/ml). **E.** and **F.** Boyden chamber assay for MDA-MB-231 cells that were transiently transfected with N-Ras siRNAs, Lin28b siRNAs, mock or GFP siRNA, and then plated on the 8 μm cell culture inserts coated with **F.** or without **E.** matrigel, and exposed to CCL18 (20 ng/ml). **C.**, **D.**, **E.** and **F.** Representative images of invasion and migration are shown; all images were obtained at 100X magnification under an inverted microscope. The cells that had migrated were counted from ten randomly chosen fields. *P > 0.05, ^#^*P* < 0.05 and ^##^ *P* < 0.01 compared with the untreated cells. Error bars correspond to the mean ± S.D. of triplicate experiments.

To determine the role of miR98 and miR27b in CCL18-promoted breast cancer metastasis, we transfected miR98 or miR27b mimics or their negative controls into MDA-MB-231 or MCF-7 cells, and determined by qRT-PCR that the reduction of miR98 or miR27b was reversed by the miRNA mimics (data not shown). Interestingly, immunofluorescence staining showed that the reduced E-cadherin and increased vimentin, which were induced by CCL18 as previously reported, were alleviated in the MCF-7 breast cancer cells transfected with miR98 mimics, but not with irrelevant negative controls (Figure 5A). These findings were further confirmed by western blot analysis showing that miR98 mimics could reverse CCL18-induced E-cadherin reduction and vimentin upregulation (Figure 5B). Therefore, retrieving miR98 expression could inhibit CCL18-induced EMT in breast cancer cells.

To further investigate whether miR98 or miR27b is involved in CCL18 promoted migration and invasion of breast cancer cells, we used Boyden chamber assay to measure the migration and invasion of MDA-MB-231 cells. Cells transfected with miR98 or miR27b mimics or negative controls or mock-transfected were plated on 8-μm cell culture inserts coated with or without matrigel. CCL18 treatment for 6 or 8 hr enhanced the migration and invasion of breast cancer cells that were transfected with a negative control or mock-transfected by 4- and 3-fold, respectively (Figure 5C, 5D; Figure SF, SG). However, MDA-MB-231 breast cancer cells that were transfected with miR98 or miR27b mimics had a significant reduction in their migration and invasion abilities by approximately 60% or 70%, respectively, upon treatment with CCL18 (Figure 5C, 5D; Figure SF, SG). These data suggest that retrieving miR98 or miR27b expression in breast cancer cells may alleviate CCL18-promoted migration and invasion of breast cancer cells.

Next, we further evaluated whether CCL18 promoted migration and invasion of breast cancer cells could be inhibited by N-Ras or Lin28b suppression. MDA-MB-231 cells were transfected with siRNAs for N-Ras or Lin28b, and were then plated on the 8-μm cell culture inserts of Boyden chambers coated with or without matrigel. After 6 hr or 8 hr of exposure to CCL18, MDA-MB-231 cells that were mock-transfected or transfected with GFP-siRNA demonstrated an increase in migration and invasion by 4-fold and 3-fold, respectively, compared with the untreated group. By contrast, cells that were transfected with N-Ras or Lin28b siRNAs significantly lost their invasion and migration ability by approximately 70% when they were treated with CCL18 (Figure 5E, 5F). These data suggest that knocking down N-Ras or Lin28b may recapitulate the effect of retrieving miR98 expression to suppress CCL18-promoted migration and invasion of breast cancer cells.

### MiR98 reduction contributes to CCL18 enhanced breast cancer metastasis *in vivo*

**Figure 6:**
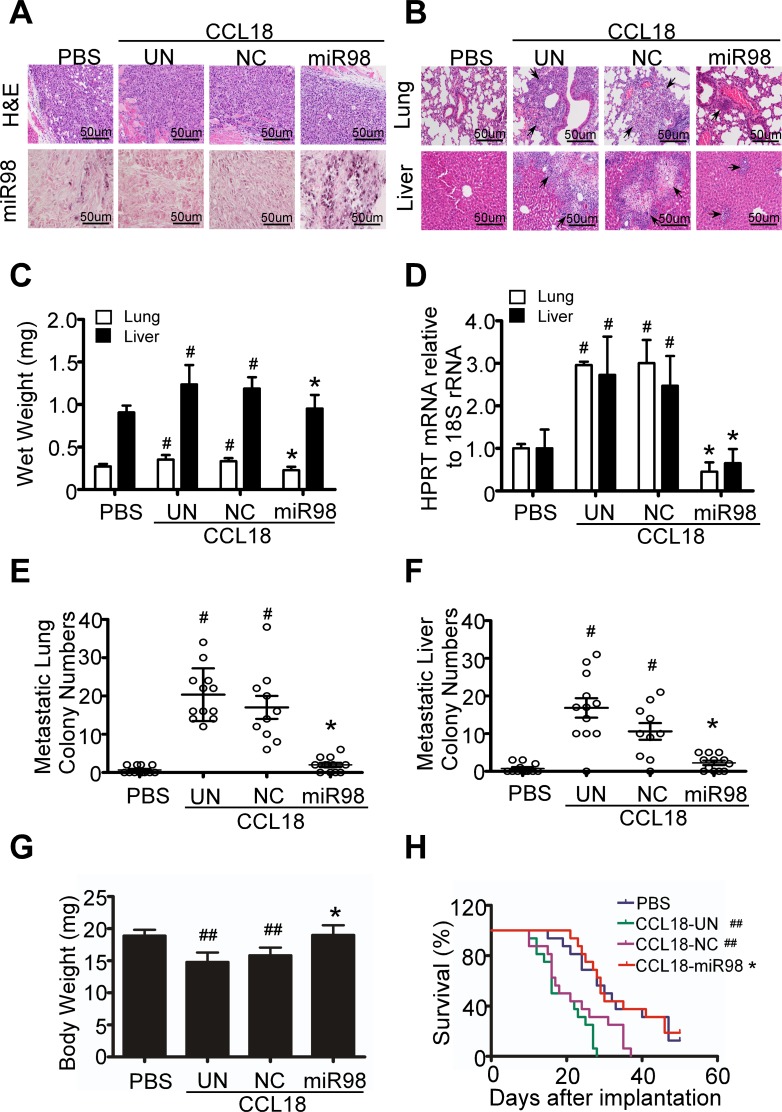
CCL18 decreases miR98 level to enhance the invasion and metastasis of breast cancer *in vivo* **A.** Microscopic images of H&E staining (upper) and ISH of miR98 (below) in the breast cancer xenografts of MDA-MB-231 cells. ISH positive signals stained blue in the cytoplasm. **B.** H&E staining of the lungs and livers of mice with MDA-MB-231 xenografts. **C.** Wet weight of the lungs and livers of mice with MDA-MB-231 xenografts. **D.** Expression of human HPRT mRNA relative to mouse 18S rRNA in the lungs and livers of tumor-bearing mice. **E.** and **F.** Quantification of metastatic tumor nodules in the lungs **E.** and livers **F.** of mice with MDA-MB-231 xenografts. **G.** Body weight of tumor-bearing mice. **C.**, **D.**, **E.**, **F.** and **G.** show *P > 0.05, ^#^*P* < 0.05 and ^##^*P* < 0.01 compared with the PBS treatment group. Bars correspond to the mean ± S.D. of triplicate experiments. **H.** Kaplan-Meier survival curve for mice with MDA-MB-231 xenografts. *P > 0.05 for comparison of the CCL18-miR98 group with the PBS group. ^##^*P* < 0.01 compared with the CCL18-UN group and the CCL18-NC group with the PBS group.

To evaluate whether miR98 reduction contributes to CCL18 enhanced breast cancer metastasis *in vivo*, we inoculated MDA-MB-231 breast cancer cells that were infected with lentivirus carrying NC or miR98 and stimulated by CCL18 (20 ng/ml) for 14 d into the mammary fat pads of nude mice. When the xenografts reached a diameter of 5 mm, we performed intra-tumoral injection of CCL18 at a dosage of 0.1 μg/kg biweekly for 25 d and evaluated tumor metastasis to the lungs and livers 50 days after inoculation. *In situ* hybridization (ISH) for miR98 in the tumor xenografts demonstrated that miR98 expression was significantly lower in the CCL18-treated animals carrying uninfected tumors or tumors infected with lentivirus carrying NC mimics than in the PBS-treated ones. However, CCL18 treatment did not result in miR98 reduction in the tumor xenografts infected with lenti-miR98 (Figure 6A). In agreement with our previous study, CCL18 injection led to increased metastasis in the lungs and livers of the nude mice carrying MDA-MB-231 xenografts that were uninfected or infected with lenti-NC virus, as determined by H&E staining, average wet organ weight and counting of metastatic colonies in the lungs and livers (Figure 6B, 6C, 6E, 6F). Additionally, quantification of human hypoxanthine phosphor-ribosyl transferase (HPRT) mRNA also demonstrated that CCL18 increased the number of metastatic breast cancer cells in the lungs and livers of the xenografted mice by 3-fold (Figure 6D). However, CCL18 failed to increase the wet weight, metastasis formation and expression of human HPRT mRNA in the lungs and livers of the animals inoculated with MDA-MB-231 cells infected with lenti-miR98 (Figure 6B, 6C, 6D, 6E, 6F). Moreover, lenti-miR98 infection abrogated the weight loss (Figure 6G) and prolonged the survival of tumor bearing mice treated with CCL18 (Figure 6H). Collectively, these data suggest that reduction in miR98 contributes to CCL18 promoted breast cancer metastasis *in vivo*.

## DISCUSSION

In the microenvironment of solid tumors, tumor associated macrophages (TAMs), which are key components of the inflammatory circuits, promote tumor metastasis by interacting with cancer cells via secretion of growth factors, angiogenic mediators, extracellular matrix-degrading enzymes and inflammatory cytokines [18, 19]. When TAM-derived inflammatory cytokines promote the metastasis of tumor cells to secondary sites through binding to their receptors, the expression of some miRNAs in tumor cells is deregulated [20, 21]. We previously demonstrated that CCL18, which is a characteristic C-C chemokine and an inflammatory cytokine that is secreted by alternatively activated M2 macrophages, promotes breast cancer metastasis and is inversely correlated with patient prognosis [3]. Our present study further reveals that M2 macrophages can reduce the expression of miR98 and miR27b in breast cancer cells through the secretion of CCL18.

MiR98 is a member of the let-7/miR98 family of miRNAs, which are often lost in less differentiated human cancer cells and cancer stem cells. These miRNAs are tumor suppressors by targeting oncogenes including Ras, HMGA2 and MYC [22]. Of note, miR98 inhibits cell proliferation, survival, growth, invasion and angiogenesis in breast cancer [23]. Subsequent studies have shown that the expression of miR98 is significantly decreased in solid tumors such as nasopharyngeal carcinoma and head and neck squamous cell carcinoma [24, 25]. Although the biological functions of the let-7/miR98 family have been studied extensively, we extend current knowledge by showing that miR98 is significantly lower in human invasive breast cancer tissues compared with benign breast tissues, which is inversely correlated with the infiltration of CCL18-positive TAMs in invasive breast cancer [3]. *In vivo*, miR98 represses CCL18-promoted metastasis and enhances the survival rate of mice with breast cancer xenografts. Therefore, down-regulation of miR98 contributes to CCL18 related breast cancer progression.

In addition, previous studies have shown that miR-27b expression is much lower in breast cancer than the adjacent noncancerous breast tissue [10, 26]. Moreover, microRNA arrays have demonstrated that miR27b is significantly lower in highly invasive breast cancer cells than the noninvasive ones [11]. In agreement, our present study indicates that CCL18-induced miR27b reduction in both MDA-MB-231 and MCF-7 cells promotes breast cancer invasion and migration.

Epithelial mesenchymal transition (EMT) is a biological process in which polarized epithelial cells gain invasive properties upon transforming into mesenchymal cells by losing tight cell-cell junctions and gaining enhanced migratory capacity [27, 28]. EMT of tumor cells could be triggered by extrinsic inflammatory stimulation and maintained by activation of intrinsic pathways that endow the cancer cells with invasive and metastatic ability [29-31]. It is well established that CCL18 induces EMT in breast cancer cells by activating the Raf/FAK/MAPK, PI3K/AKT and NFκB pathways [3, 4, 32, 33]. Ras activation plays a crucial role in the development of human malignancies by promoting EMT and metastasis [32, 34-36]. After exposure to CCL18 for 12 hr, the N-Ras/ERK/PI3K/AKT signaling pathway in breast cancer cells is activated and triggers activation of NFκB, which is the central mediator for the maintenance of EMT *in vivo* and *in vitro*. Therefore, inhibition of NFκB could reverse EMT in Ras-transformed mammary epithelial cells [37]. It has been verified that activation of NFκB directly increases Lin28b expression, which subsequently decreases let-7a expression in Scr-activated mammary cells [17]. We have also observed that lin28b overexpression is induced following NFκB activation after exposure to CCL18 for 12 hr. After treatment with CCL18, the N-Ras/ERK/PI3K/NFκB/Lin28b pathway was consistently active, which was maintained by down-regulation of miR98 and miR27b, leading to EMT of epithelial breast cancer cells.

Epigenetic switch requires an initiating event, such as an inflammation-induced signal, but the phenotype of the mesenchymal cells could be maintained by self-sustained feedback loops, even when the initial signal is absent [38]. Inflammatory chemokines favor the activation of EMT inducers including NFκB, SNAIL, STATs, and SMADs [39]. Such activation causes changes in the expression of miRNAs that in turn regulate the effects of chemokine signaling by targeting different signal factors. Together, the chemokines themselves or the modulators of chemokine signaling further regulate EMT in epithelial cells [28]. MiRNAs directly or indirectly target EMT signaling factors, forming feedback loops, because they themselves are regulated by transcription factors [36]. Our study is the first to demonstrate a positive feedback loop between CCL18 and miR98. Because the N-Ras-3′-UTR contains a putative binding site of miR98, N-Ras is the target of miR98 in breast cancer cells. Some studies have revealed that the expression of certain members of the let-7/miR98 family is regulated at both the transcriptional and post-transcriptional levels [40]. Our data indicate that miR98 was regulated at post-transcriptional level by lin28b. Lin28b is a family of RNA binding proteins and microRNA regulators, which is involved in a potential regulatory feedback loop with miR122 via the activation of c-Myc in prostate cancer [41]. Moreover, Lin28b binds to the terminal loops of the precursors of let-7/miR98, which blocks the processing of let-7/miR98 into its mature form by Dicer and Drosha. This is important for the coordinated inhibition of all let-7 family members in mammalian cells [42, 43]. When N-Ras is silenced, the entire ERK/PI3K/NFκB/Lin28b pathway is inactivated and miR98 loses its inhibitor Lin28b, resulting in higher miR98 level to further inhibit N-Ras expression. When Lin28b is knocked down, mature miR98 is released to target N-Ras directly and then suppress the ERK/PI3K/NFκB/Lin28b pathway. Therefore, activation of the N-Ras/ERK/PI3K/NFκB/Lin28b pathway is responsible for down-regulation of miR98 and thus enables abundant N-Ras expression to sustain active CCL18 signaling. A positive feedback loop between CCL18 pathway and miR98 down-regulation was then formed to maintain EMT and promote migration and invasion of breast cancer cells.

On the other hand, miR-27b expression has been associated with enhanced E-cadherin expression and reduced metastatic characteristics of breast cancer cells [44]. miR27b is linked to multiple EMT regulatory pathways, including PI3-kinase, MAPK, TGFβ, WNT, mTOR, JAK-STAT, and Notch [45]. In our present study, miR-27b abrogated CCL18-induced migration and invasion of breast cancer cells by silencing Lin28b, and led to increased mature miR98 to block CCL18 signaling. Therefore, CCL18 induced down-regulation of miR-27b contributes to maintain low miR98 level and active CCL18 signaling. Although previous studies have shown that the MAPK and NFκB pathways inhibit miR27b expression [8, 45], the mechanism for CCL18 to dampen miR27b expression is still unclear.

In summary, our study shows that CCL18, an important TAM-derived cytokine, can repress the expression of miR98 and miR27b in breast cancer cells. In addition, miR98 and miR27b participate in a positive feedback loop that sustains the CCL18-activated N-Ras/ERK/PI3K/NFκB/Lin28b signaling pathway, which could be a potential therapeutic target to block CCL18 function.

## MATERIALS AND METHODS

### Cell culture, treatment and transfection

MDA-MB-231 and MCF-7 breast cancer cells were obtained from American Type Culture Collection (ATCC, USA). The detailed procedure of how cell culture, treatment and transfection are carried out has been described in previous study [3].

### Patients and tissue samples

Primary ductal carcinomas of the breasts were obtained from 60 female patients at the Sun Yat-Sen memorial Hospital, Sun Yat-Sen University, from January 2005 to December 2008. All samples were collected with informed consent from the Internal Review and the Ethics Boards of the Sun Yat-Sen memorial Hospital, Sun Yat-Sen University.

### MiRNA array analysis

The miRNA expression changes between the MDA-MB-231 parental cells co-cultured with M2 macrophages or treated by CCL18 (PeproTech, USA) and the untreated ones were analyzed using Generation III array scanner (Amersham Pharmacia Biotech, USA) under the manufacturer's recommendations.

### MicroRNA *in situ* hybridization

ISH of miRNA was performed according to a previous study [46]. Digoxigenin-labeled locked nucleic acid probes of miR98 (probe concentration 80 nM), U6 (positive control; probe concentration 40 nM) and scrambled RNA (negative control; probe concentration 40 nM) was purchased from Exiqon (USA).

### Cell migration and invasion assay

Migration and invasion assays were carried out using 24-well Boyden chambers (Corning, USA) with 8M-inserts coated with fibronectin (Roche, USA) and Matrigel (BD Biosciences, USA) as described previously [3].

### RNA extraction and qRT–PCR

Detailed procedures of RNA extraction and qRT–PCR were described elsewhere [3]. Specific primers used in this experiment were described in Supplementary Table 1.

### Western blotting

Detailed procedure was described as previously [3].

### Immunoﬂuorescence staining and microscopy

Detailed procedure was described as previously [16].

### Antibodies and reagents

Detailed information of antibodies and reagents used in this study is provided in Supplementary Table 2.

### 3′-UTR luciferase reporter assay

Human N-Ras 3′-UTR (3630 bp) containing the putative binding sites of miR98 and Lin28b 3′-UTR (4548 bp) with the putative binding sites of miR27b were amplified by PCR, inserted into the firefly luciferase reporter vector pmiR-RB-Reporter^™^ (pWT, RiboBio Co. Ltd, China) between the restrictive sites Xho I and Not I, and validated by sequencing. Its mutant constructs (N-Ras or Lin28b 3′-UTR,pMut) with a mutation of the miRNA (miR98 or miR27b) binding site was generated with the mutagenic oligo nucleotide primers (Supplementary Table 1), according to the manual of GeneTailor Site Directed Mutagenesis System (Invitrogen). The 293T cells were transfected with miR-mimics (RiboBio Co. Ltd) and pWT, or miR-mimcs and pMut, or miR-negative control (nc) and pWT, or miR-nc and pMut. Cells were harvested after 24 hr and the luciferase activity was assayed according to the dual-luciferase assay manual (Promega, USA). The Renilla luciferase signal was normalized to the firefly luciferase signal for each individual analysis.

### Tumor xenografts

Female athymic nude mice were bred and maintained under defined conditions at the Animal Experiment Center of Sun-Yat-Sen University. All procedures were approved by the Animal Care and Use Committee of Sun Yat-Sen University and conformed to the legal mandates and national guidelines for the care and maintenance of laboratory animals. Breast cancer MDA-MB-231 cells, which were uninfected or infected with lentivirus carrying NC or miR98 analogue, were stimulated by CCL18 (20ng/ml) for14 days, and then inoculated into the mammary fat pads of the mice (*n* = 16/group). When xenografts were palpable (around 5mm in diameter), intra-tumor injection of CCL18 was performed at a dosage of 0.1 ug/kg biweekly for 25 days. When the xenografts reached 1.5cm in diameter in about 50th days, the animals were sacrificed and the tumor xenografts, lungs, and livers of the mice were harvested for further evaluation. MicroRNA *in situ* hybridization and H&E for miR98 detection or histological assessment was performed on paraffin sections (4 mm) of the xenografts or harvested organs, and total RNA was extracted from the mice organs for qRT-PCR analysis of human HPRT mRNA expression.

### Statistics

All experiments for cell cultures were performed independently for at least three times and in triplicate for each time. Data were presented as mean±SD. Student's t test and one-way ANOVA were used to correlate miR98 level with breast cancer metastasis, whereas Kaplan-Meier method was used to estimate survival. All *P* values were two-tailed and considered significant at less than 0.05. All statistical analyses were performed using SPSS for Windows version 16.0 (SPSS, Chicago) and GraphPad Prism 5.01.

## SUPPLEMENTARY FIGURES AND TABLES


