# Supplementary material for: CCL18-mediated down-regulation of miR98 and miR27b promotes breast cancer metastasis
Source: Oncotarget. 2015 May 12;6(24):20485–99. doi: 10.18632/oncotarget.4107 (PMC4653020; doi:10.18632/oncotarget.4107)
Supplement: Supplementary file 1 [file oncotarget-06-20485-s001.pdf]

CCL18-mediated down-regulation of miR98 and miR27b promotes breast cancer metastasis

Supplemental Information

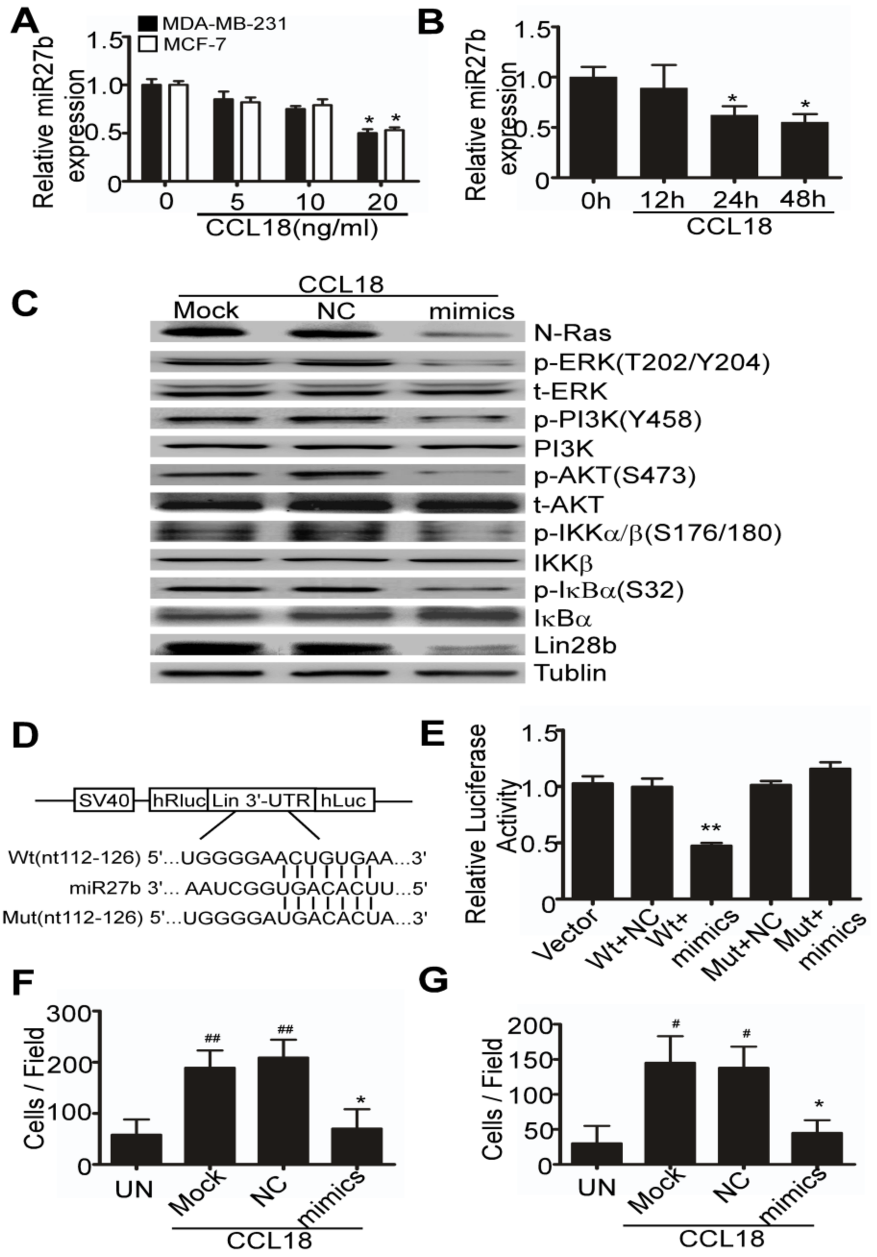

Figure Supplemental: CCL18 reduces miR27b to sustain N-Ras/ ERK/ PI3K/NFκB/Lin28b pathway, promotes migration and invasion of breast cancer

(A)The ratio of miR27b normalized to U6 in breast cancer MDA-MB-231 and MCF-7

cells, treated with different concentrations of CCL18 for 24hr, as assayed by qRT-PCR.

(B)qRT-PCR analysis alterations of miR27b expression in MCF-7 cells untreated (0 hr) or treated with CCL18 (20ng/ml) for different time points.

For (A)(B), **P*<0.05 as compared to the untreated cells. Data are expressed as mean ± SD of triplicates.

(C)Western-blot analysis for the expression of the phosphorylated and total proteins of N-Ras, ERK, PI3K, AKT, IKKα, IKKβ, IκBα, and Lin28b in MCF-7 cells transfected with or without miR27b mimics; tublin was used as a loading control .

(D)The miR27b targeting sequence located in the 3'-untranslated region of Lin28b.

(E)MiR27b decreased Lin28b luciferase activity. The reporter plasmids (pmiR-control, pmiR-Lin28b-Wt, and pmiR-Lin28b -Mut) were transiently transfected into 293T cells, along with a hRluc/hluc expression plasmid. Luciferase activities were measured after 24 hr and normalized against hluc values. The data were the means±S.D. of separate transfections (n=3) and were shown as the ratio of renilla reniforms activity to firefly luciferase activity. The percentage of relative luciferase (RLU) activity was plotted ***P* <0.01

(F and G)Boyden chamber assay for the MDA-MB-231 cells that were transiently transfected with miR27b mimics, negative control or mock , plated on the 8um cell culture inserts coated with (G) or without (F) matrigel, and treated with medium only or CCL18 (20ng/ml). Representative photos of migration (F) and invasion (G) are taken at 100X magnification under an inverted microscope. The migrated cells

were counted from ten randomly chosen fields. *P>0.05, #P<0.05 and ## P< 0.01 compared with the untreated cells. Error bars correspond to mean ± SD of triplicates.

Supplementary Table 1. The list of primers and oligomers used in this study.

Oligomers	Sequences
Human miR98 forward primer	5’-TGTATGACTGCCGTATGTTTCCTATT-3’
Human miR98 reserve primer	5’-AATTCTTAAAGTATGCTTTCCATTCC-3’
Human miR27b forward primer	5’-ACACTCCAGCTGGGTTCACAGTGGCTAAG-3’
Human miR27b reverse primer	5’-CTCAACTGGTGTCGTGGAGTCGGCAAT-3’
Human miR20a primer	5’-GCCGCGCTAAAGTGCTTATAGTG-3’
Human miR20a primer	5’-CACCAGGGTCCGAGGT-3’
Human U6 forward primer	5’- CTCGCTTCGGCAGCACA-3’
Human U6 reverse primer	5’- AACGCTTCACGAATTTGCGT-3’
N-Ras forward primer	5’- ATGAGGACAGGCGAAGGCT-3’
N-Ras reverse primer	5’- TGAGTCCCATCATCACTGCTG-3’
Lin28b forward primer	5’- GGATTTGGATTCATCTCCATGATAA-3’
Lin28b reverse primer	5’- GAATTCCACTGGTTCTCCTTCTTTT-3’
Human GAPDH forward primer	5’- GTGGACCTGACCTGCCGTCT-3’
Human GAPDH reverse primer	5’- GAGGAGTGGGTGTCGCTGT-3’
CCL18 si-RNA 1	Sense 5′-ACAAGTTGGTACCAACAAATT-3′<br>Antisense 5′-TTTGTTGGTACCAACTTGTGC-3′
CCL18 si-RNA 2	Sense 5′-CCAGCATTCTCACTGTGAATT-3′<br>Antisense 5′-TTCACAGTGAGAATGCTGGTT-3′
N-Ras si-RNA 1	Sense 5’-GCACTGACAATCCAGCTAATT-3’<br>Antisense 5’-TTAGCTGGATTGTCAGTGCTT-3’
N-Ras si-RNA 2	Sense 5’-GCAAGTCATTTGCGGATATTT-3’<br>Antisense 5’-ATATCCGCAAATGACTTGCTT-3’
Lin28b si-RNA 1	Sense 5’-CACCAAAGCAAACTATTCATT-3’<br>Antisense 5’-TGAATAGTTTGCTTTGGTGTT-3’
Lin28b si-RNA 2	Sense 5’-GGAGATAGATGCTACAACTTT-3’<br>Antisense 5’-AGTTGTAGCATCTATCTCCTT-3’
GFP si-RNA	Sense 5’-GGCTACGTCCAGGAGCGCACC-3’<br>Antisense 5’-TGCGCTCCTGGACGTAGCCTT-5’

Oligomers	Sequences
HPRT forward primer	5′-TTCCTTGGTCAGGCAGTATAATCC -3′
HPRT reverse primer	5′-AGTCTGGCTTATATCCAACACTTCG -3′
18S rRNA forward primer	5′- CGGCTACCACATCCAAGGAA -3′
18S rRNA reverse primer	5′- GCTGGAATTACCGCGGCT -3′
N-Ras Wt 3’-UTR forward primer	5'-CCGCTCGAGTAACTACCTCCTCACTTGGCTGT-3'
N-Ras Wt 3’-UTR reverse primer	5'-GAATGCGGCCGCAAGAATTATGACTAAGCCAAGAAC-3'
N-Ras mut 3’-UTR forward primer	5'-CCGCTCGAGTAAGATGCTGCTCACTTGGCTGTC-3'
N-Ras mut 3’-UTR reverse primer	5'-GAATGCGGCCGCAAGAATTATGACTAAGCCAAGAAC-3'
Lin28b Wt 3’-UTR forward primer	5'- GCGGCTCGAGGTTCTTTCCTTTACCCGGTT-3'
Lin28b Wt 3’-UTR reverse primer	5'-AATGCGGCCGCCCTCCCCTTCATTTCTCTTA-3'
Lin28b mut 3’-UTR forward primer	5'-ATTGGGGATGACACTTTTTTTTAAACAGACAAATC-3'
Lin28b mut 3’-UTR reverse primer	5'-TTAAAAAAAAGTGTCATCCCCAATAGTAGTTAAAA-3'

Supplementary Table 2. Information of antibodies and reagents.

Western blotting	
	Anti-human E-cadherin(CST , Danvers, MA, 1:1,000)<br>Anti-human vimentin(R&D system,Minneapolis,MN, 1:1,000)<br>Anti-human p-ERK(1/2) (CST,1:2,000)<br>Anti-human ERK(1/2) (CST,1:1,000)<br>Anti-human p-AKT(S473) (CST, 1:2,000)<br>Anti-human AKT (CST,1:1,000)<br>Anti-human p-PI3K(Y458) (CST,1:1,000)<br>Anti-human PI3K (CST,1:1,000)<br>Anti-human p-IKKα/β(S176/180) (CST,1:1,000)<br>Anti-human IKKα(CST,1:1,000)<br>Anti-human IKKβ(CST,1:1,000)<br>Anti-human p-IκBα(S32) (CST,1:1,000)<br>Anti-human IκBα(CST,1:1,000)<br>Anti-human Lin28b (CST,1:1,000)<br>Anti-human N-Ras(Abgent, Santiago,CA, 1:500)<br>Anti-human Tublin(Sigma-Aldrich, St Louis, MO, 1:10,000)<br>Anti-human GAPDH (CST,1:3,000)<br>Peroxidase-conjugated anti-mouse or rabbit IgG (CST, 1:10,000)<br>Enhanced chemiluminescence assay(ECL)(Thermo, Rockford, IL)
Chemicals	
	Interleukin 4(IL-4)(R&D,USA)<br>CCL18 neutralized antibody(Abcam,USA)<br>Isotype-matched IgG (Abcam)<br>Lipofectomine((Invitrogen,USA)<br>Trizol (Invitrogen)
QRT-PCR	
	One Step SYBR PrimeScript™ RT-PCR Kit (Perfect Real Time)(Takara,China)<br>TaqMan Pri-miRNA Assays(Applied Biosystems,USA)
Immunofluorescence	
	Anti-human E-cadherin (CST,1:100)<br>Anti-human vimentin(R&D system,1:50)<br>DAPI (Sigma)<br>Alexa Fluor-488 Chicken Anti-Rabbit IgG (Invitrogen,1:250)<br>Alexa Fluor-594 Chicken Anti-Rat IgG (Invitrogen,1:250)<br>confocal laser scanning microscopy (LSM710, Zeiss, Germany)
